# Parental/caregiver racial-ethnic socialization during early childhood: a meta-ethnographic review

**DOI:** 10.3389/fpsyg.2026.1716005

**Published:** 2026-02-04

**Authors:** Kaitlin N. Quick, Julia Mendez Smith, Stephanie Irby Coard, Michaeline Jensen

**Affiliations:** 1 Department of Psychology, University of North Carolina at Greensboro, Greensboro, NC, United States; 2 Department of Human Development and Family Studies, University of North Carolina at Greensboro, Greensboro, NC, United States

**Keywords:** early childhood, ethnic-racial socialization (ERS), parenting, race, racial-ethnic socialization (RES)

## Abstract

**Introduction:**

Racial-ethnic socialization (RES) is an ongoing developmental process through which parents and caregivers share information, values, and perspectives about race and ethnicity with their children. While there is a substantial body of research on RES during adolescence, little empirical work has focused on early childhood.

**Methods:**

This review applied a systematic search strategy and meta-ethnographic methods to examine and integrate the existing qualitative research on RES during early childhood. Out of 1,900 screened studies, 277 were reviewed for eligibility, and 27 met the inclusion criteria.

**Results:**

Findings from the seven phase meta-ethnographic review process highlight the dynamic and multifaceted nature of RES in parenting. Parents' own life experiences strongly shape their values, beliefs, and intentions for RES, which in turn inform the approaches and messages they use with their children. These strategies are often interrelated but can also be influenced by competing beliefs triggered by children's characteristics, experiences, and environments.

**Discussion:**

Overall, the synthesis supports prior claims that RES in early childhood is deeply embedded within general parenting practices and family dynamics. It also identifies critical gaps in the literature (e.g., limited diversity of study samples in terms of family structures and racial-ethnic backgrounds) that future research should address.

## 1 Introduction

Parental/caregiver racial-ethnic socialization (RES) is a lifelong developmental process through which parents/caregivers transmit “information, values, and perspectives about race and ethnicity” to their children (Hughes et al., 2006). Parental/caregiver RES during early childhood lays the foundation for future identity development and the development of racial attitudes (Castelli et al., 2009; Williams et al., 2020b), as early childhood is a formative period marked by rapid cognitive, social, and emotional development during which children begin to understand race/ethnicity as a salient construct. While there is a growing body of literature examining RES during adolescence, there is little empirical evidence about RES processes during early childhood.

### 1.1 Racial-ethnic socialization

Out of the first studies conducted in the 1980s seeking to identify normative parenting behaviors of African American families, the construct of RES began to take form in the literature (Peters and Massey, 1983; Spencer, 1983). One of the earlier conceptualizations of RES described a “triple quandary” of tasks facing African American parents—promotion of cultural pride, preparation for mainstream society, and preparation for coping with racism and discrimination (Boykin and Toms, 1985). From the 1990s into the early millennium researchers' interest in RES largely focused on examining RES messaging and behaviors across different racial-ethnic groups, and on identifying correlates, precursors, and outcomes of these processes (Brown et al., 2007; Hughes, 2003; Thornton et al., 1990). It has been posited that this focus was at least partially driven by a push to contextualize child development within an ecological perspective (McLoyd, 2006).

In 2006, (Hughes et al. 2006) published what most consider to be the first comprehensive review of the RES literature, identifying four common components across studies (cultural socialization, preparation for bias, promotion of mistrust, and egalitarian socialization). Cultural socialization—or “pride messages”—are the ways parents expose their children to histories, traditions, and values of their culture as well as promote belonging to- and pride in membership of their cultural group. Preparation for bias encompasses the ways parents promote awareness of- and coping with potential threats such as discrimination and racism. Promotion of mistrust describes messages and practices that promote wariness of other racial-ethnic groups—often the majority group (i.e., White) but doesn't necessarily include advice for coping. Finally, egalitarian socialization is defined as practices that center on the idea of racial equality by emphasizing the development of skills, attitudes, and characteristics that facilitate success within the dominant majority culture. One of the lasting effects of this paper was giving the field a common vernacular to describe these processes across groups.

An updated review of the RES literature moved beyond examination of RES as a broad construct and evaluated the evidence for the four types of messages separately (Umaña-Taylor and Hill, 2020). Authors found that most studies examined cultural socialization messaging and provided strong evidence of associations with positive youth outcomes such as ethnic-racial identity, self-esteem, academic adjustment, and overall psychosocial wellbeing. Evidence for the other three types of messages was sparse; however, they found a trend toward positive associations between egalitarianism and positive youth outcomes in majority Black samples, robust negative outcomes for promotion of mistrust, and mixed evidence for preparation for bias messages that may indicate dependence on context such as parenting or family relationship.

One limitation of these findings is that most studies examine these processes primarily in adolescent samples, with the least studied developmental period being early childhood. Umaña-Taylor and Hill's (2020) systematic review found that only 19% of 259 identified studies included “childhood” aged samples, and a 2014 systematic review found that only 15% of 92 identified studies focused on the early childhood period (Priest et al., 2014). This focus on adolescence may be due to researchers' interest in associations with other racial/ethnic processes that are commonly studied in adolescence such as identity development and discrimination. It has also been posited that the discrepancy may be due to the enduring impact of early study results indicating that parents were less likely to have discussions about race with their younger children (Ruck et al., 2021).

### 1.2 Development of racial awareness and biases during early childhood

This review argues for a renewed examination of RES in early childhood, given that early childhood is an important developmental period during which rapid cognitive growth promotes awareness and abilities to categorize, with race/ethnicity emerging as a salient category by which children begin to base decisions and behaviors. Studies have shown that around 3 months of age, infants develop preferences for faces from their own ethnic group (Kelly et al., 2005). By 6 months differences in the salience of race vs. gender cues have been observed when comparing Black and White infants, with race being more salient for Black infants than White (Katz, 2003). By 9 months infants are able to racially categorize and group faces based on differential experience/exposure to other races (e.g., infants broaden the distinction between same and other race faces forming a larger “out-group” category; Anzures et al., 2010; Quinn et al., 2016). All of this suggests that within the first year of life infants are beginning to develop preverbal concepts of race/ethnicity or racial/ethnic “awareness.”

Between 18- and 30-months children show steady increases in their abilities to accurately sort dolls and pictures by race and begin attempts to self-label their own race (Katz and Kofkin, 1997). Between 36 and 48 months children's racial classification abilities drastically increase in tandem with evidence of developing racial bias (e.g., white/light bias in doll selection, and associating anger with Black faces; Dunham et al., 2013; Gibson et al., 2015). Then between the ages of 4 and 6 years, skin-tone emerges as one of the most salient features in determining similarities between faces (Balas et al., 2015). A multinational meta-analysis found that children's prejudices toward (racial, ethnic, and national) outgroups peaks between the ages of 5 and 7 years and subsequently declines (Raabe and Beelmann, 2011). Around the same age children also begin to perceive unfair treatment of others as racial/ethnic discrimination (Brown, 2006).

Despite strong evidence for these early race-related developmental milestones, a recent study suggests that on average American adults believe that first discussions about race should occur around the age of five (Sullivan et al., 2021). Authors additionally found that adults misjudged the ages at which basic racial developmental processes occurred (e.g., developing racial awareness and racial biases) by approximately 4.5 years, and that these inaccurate beliefs were the strongest predictor of the age at which adults were willing to have conversations about race and racism with their children. In other words, because American adults inaccurately believe that children younger than five aren't aware of race and racial biases, they don't talk to their young children about race and racism.

### 1.3 Rates of RES during early childhood

In line with these findings, there has been consistent evidence that explicit *discussions* about race and ethnicity increase in frequency across different racial/ethnic populations as children age (Hughes et al., 2006). (Hughes and Chen 1997) found relatively linear increases in Black families' use of preparation for bias and promotion of mistrust messaging between age groups from 4 to 14 years, and that levels of cultural socialization were relatively high and stable across age groups until pre-adolescence. A study of adoptive families similarly found that children's adoptive parents increased their use of preparation for bias messages between the ages of four and 14; however, while cultural socialization messages increased between the ages of four and 10 they were at much higher levels to begin with (Johnston et al. 2007). More recently a longitudinal study of Black and Latine preschoolers found that parents' use of cultural socialization and preparation for bias messages increased between ages 2.5 and seven, with a sharp increase around kindergarten entry (Contreras et al., 2022).

This is not to imply that RES does not occur at substantial rates during early childhood. Approximately 56% of the 17,372 parents of kindergarteners in the Early Childhood Longitudinal Study 1998–1999 Kindergarten entry cohort (ECLS-K) reported discussing race/heritage with their kindergartener several times a year or more (Lesane-Brown et al., 2010). Studies examining rates of specific types of messages in particular groups have found cultural socialization to be the most prevalent RES strategy during early childhood (Aguayo et al., 2021; Caughy et al., 2002; Contreras et al., 2021, 2022; Johnston et al., 2007; Lee et al., 2006; Tyrell et al., 2023).

Evidence regarding prevalence of other messages during early childhood is mixed. Egalitarian messages were the most prevalent in one study of low-income racially diverse parents of children enrolled in Head Start (Davidson and Roopnarine, 2022). Egalitarian and color-blind messaging have been shown to be especially prevalent in White families raising White children (Vittrup, 2018). Although preparation for bias messages were initially thought to be used relatively infrequently during early childhood, several studies have shown that they are also prevalent—especially in Black families (Caughy et al., 2002; Contreras et al., 2022; Davidson and Roopnarine, 2022; Tyrell et al., 2023), and to a slightly lesser extent among Latine parents (Contreras et al., 2022). Across racial-ethnic groups, promotion of mistrust is the least frequently endorsed type of RES that parents of young children engage in (Caughy et al. 2002), (Hughes and Chen 1997), and (Tyrell et al. 2023).

### 1.4 Factors associated with early childhood RES

#### 1.4.1 Child traits

Racial identity of the child has been shown to predict RES practices during early childhood. Non-White—specifically Black—racial identity has been linked with increased overall frequency of RES discussions (Brown et al., 2007; Tyrell et al., 2023). Similarly, using a Black-White biracial subsample of the ECLS-K 1998–1999 cohort data, (Csizmadia et al. 2014) found that how parents racially *identified* their biracial child predicted frequency of discussions, with children identified as White by their parents having less frequent RES discussions than children identified as Black or Biracial. Evidence of the influence of child gender on RES is mixed with studies providing evidence both for and against the influence of child gender on RES frequency (Brown, 2006; Tyrell et al., 2023). This mixed evidence could be indicative of more complex relationships between gender, other factors, and RES. For instance, (Derlan et al. 2017) found an interaction between skin tone and gender such that Mexican mothers' cultural socialization efforts and the results of these efforts varied by gender, skin tone, and interactions between the two constructs.

#### 1.4.2 Environmental factors

Contextual factors have also been shown to influence RES practices during early childhood. In a sample of unhoused families living in an emergency shelter, authors found the number of children in the family was positively associated with cultural socialization and preparation for bias messaging (Tyrell et al., 2023). This may be due to parents providing socialization messages for older children in the presence of younger children.

(Wang et al. 2022) combined data from minoritized families in the ECLS-K 1998–1999 and 2010–2011 cohorts to longitudinally investigate the role of environmental ethnic-racial composition and numeric marginalization on RES and social competence. They found that families engaged in more frequent RES discussions in spring when children were in more diverse school and neighborhood environments during the fall of kindergarten. They also found that RES measured in the spring of kindergarten had the strongest association with social competence during first grade when the child was in more diverse settings with fewer same race/ethnicity peers (Wang et al., 2022).

(Davidson and Roopnarine 2022) found evidence implicating RES message-matching between home and school in a small sample of Head Start parents and teachers. Parent-preschool teacher incongruence in RES beliefs and practices may have negative effects on children's socioemotional functioning. When parents engaged in low levels of cultural socialization while teachers engaged in high levels, children showed lower levels of initiative and self-regulation. Children with mismatched parents' and teachers' egalitarian practices had lower levels of self-regulation (Davidson and Roopnarine, 2022).

#### 1.4.3 Parent traits and experiences

Parental warmth, SES, age, and education have been shown to be associated with RES practices during early childhood (Brown et al., 2007; Hill and Tyson, 2008; Tyrell et al., 2023). Several studies have moved beyond correlations to further explore the impacts of parental RES on early childhood development. (Pahlke et al. 2012) found that even though mothers' and children's racial attitudes were statistically unrelated, mothers' cross-race friendships (i.e., exposure and modeling of diversity acceptance) predicted lower levels of children's racial bias.

In Latine families, there is preliminary evidence for intergenerational transmission of RES, with grandmothers' cultural socialization practices of teen mothers directly associated with their own cultural socialization practices with their children (Williams et al., 2020a). Mothers' own ethnic-racial identity beliefs and ethnic-racial centrality have also been linked to cultural socialization practices with their young children (Calzada et al., 2012; Derlan et al., 2017). In the same sample, authors found cultural socialization to be a protective factor against the effects of maternal risky behaviors on internalizing problems (Williams et al., 2023).

### 1.5 Gaps in the literature

A major limitation of these findings is that the most frequently cited findings are drawn from the ECLS-K 1998–1999 data set which assesses RES based on one question about the frequency of discussions about race/heritage. Most other studies utilize more comprehensive self-report measures; however, they are limited in their reliance on self-reported frequency of *verbal messages* with few questions capturing other aspects of the RES process, despite them being inextricably linked (Yasui, 2015). This is an important distinction given that parental RES processes are beginning to be conceptualized as linked rather than independent from other parental socialization processes (Dunbar et al., 2017). Additionally, other early-childhood socialization models as well as broader (e.g., not age specific) conceptualizations of parental socialization do not solely focus on verbal transmission or discussion (Zinsser et al., 2021). Emotion socialization theories often include *parental reaction* to children's emotions, and parental expression of emotion (e.g., child observation/parent modeling) in addition to direct instruction/discussion methods. Scaffolding has also been shown to be an important aspect of socialization for cultural skills that require mental processing (Gauvain, 2005). While other socialization literatures highlight the importance of these implicit methods and behavioral processes, such distinctions are not adequately captured by the current quantitative RES literature. Systematic reviews of the literature have largely focused on quantitative studies, only briefly highlighting qualitative evidence or excluding it all together. However, qualitative methods may be better situated to capture complex processes such as RES. This study aims to conduct an in-depth exploration and synthesis of the available qualitative evidence containing parent/caregiver's accounts of racial-ethnic socialization with their children under the age of six.

### 1.6 The current study

The current study uses meta-ethnographic methods to systematically examine and thematically synthesize the existing qualitative literature on parental RES during the early childhood period. We have adopted (Stokes et al. 2021)'s definition of RES which is grounded is Yasui's (2015) Process Model of Ethnic-Racial Socialization (PMERS). PMERS is based on the theory of planned behavior, and conceptualizes RES as a complex process, encompassing how parental history, environment, and beliefs lead to both explicit (e.g., intentional, conscious efforts), and implicit (e.g., sometimes unintentional behaviors, affect, or responses to their children) messages that communicate something to their children about race, ethnicity, or culture. However, given the developmental period of focus for this review, we have additionally added “*planned efforts/intentions for RES”* as extant literature has shown that large proportions of parents report not engaging in explicit discussions at this age. As such, the definition of RES used throughout all phases of the present review is as follows:

Any demonstrated or planned forms of verbal or non-verbal communication where parents convey something about the meaning, significance, and/or value of race and culture to their children including explicit (e.g., conscious and intentional efforts) and implicit messages (e.g., subtle ways that parents inadvertently teach their children about race, culture, or ethnicity through spontaneous reactions and routine practices in the family's microsystem) (Stokes et al., 2021, p. 5).

## 2 Methods

A meta-ethnographic approach was selected to synthesize this literature, as meta-ethnography aims to move beyond aggregation to inductive interpretation of qualitative evidence. The Meta-Ethnographic synthesis process established by (Noblit and Hare 1988) has become one of the most widely used synthesis methods for qualitative work (Hannes and Macaitis, 2012). Meta-ethnography is rooted in the interpretivist research paradigm. Interpretivism holds that reality is subjective and varied, and that knowledge is formed from the interactions between the researcher and participants. As such interpretivist research seeks explanation of social and cultural phenomena through the perspectives of those with lived experience and often emphasizes subjective meaning making of individual experiences. Meta-ethnography provides an alternative to positivist synthesis methods of data aggregation and seeks to inductively interpret multiple cases across studies. The goal of a meta-ethnographic review is to generate new interpretations, theories, hypotheses, or concepts from pre-existing literature while maintaining the “meaning in context” from the original source (Noblit and Hare, 1988).

### 2.1 Positionality

Given the qualitative interpretivist nature of this review, the positionality of the researchers is important to disclose as they are inextricable from the process and the findings. The first author is a cisgender Black-White Biracial woman from the southeastern United States. After her parents' divorce, she was primarily raised by her White mother. She is a sister, daughter, student, researcher, therapist, and former early childhood educator. These intersectional identities and experiences shaped her interest in studying topics at the intersection of culture, family, and early childhood. She comes to this work having previously contributed to research on parenting, race, and cross-cultural adaptations of psychosocial interventions and measures. The second author is a Mexican American woman and professor whose educational and professional path spans five universities. Her connection to early childhood began when she entered preschool at age three—attending the nursery school where her mother worked—and continued as she later enrolled her three children in various full-time childcare settings. With a career dedicated to researching Head Start and childcare programs, her experiences shape her commitment to understanding and supporting the care of young children, especially those from low-income backgrounds. The third author is an African American woman, developmentally and clinically trained psychologist, and community-engaged scholar. Her personal, professional, and academic identities shape the lens through which she approaches this work. Her lived experience as a Black mother, daughter, and community member informs both her sensitivity to the nuanced ways racial-ethnic socialization occurs within families and commitment to advancing equity in child development. Her scholarship is rooted in a culturally grounded, strengths-based framework that resists deficit narratives and acknowledges the historical and systemic contexts that influence families' practices. The fourth author is a White woman, professor, clinical psychologist, and mother to a young child. We acknowledge that our perspectives are shaped by our own cultural backgrounds and positionality, and remain committed to reflexivity, humility, and learning in ongoing collaboration with diverse communities and colleagues.

### 2.2 Search and selection criteria

In August 2024, after the authors clarified the focus of the review and synthesis, the first author conducted a comprehensive search of Academic Search Complete (EBESCO), Child Development & Adolescent Studies, PsycINFO, PsycArticles, ERIC, SocINDEX, CINAHL, JSTOR, and SCOPUS databases using specific search terms “rac^*^ socialization” OR “pride socialization” OR “preparation for bias” OR “cultur^*^ socialization” OR “ethnic socialization” OR “colorblind^*^” AND “toddler^*^” OR “young child^*^” OR “early childhood” OR “preschool^*^” OR “kindergarten^*^” to identify articles for potential inclusion in this review. The search included peer reviewed articles published in English through August 2024 and the original search yielded 802 articles. Article abstracts were imported into Covidence systematic review software for screening. Articles were screened by the first author for the following inclusion criteria: (a) empirical peer reviewed article or report (b) written in English (c) with research aim or interview question about RES (this included interview questions about parenting and race/ethnicity/culture so long as there were results about RES) (d) had a US based sample of (e) parents or primary caregivers of a child under the age of six or kindergarten and below if utilized school-based aging, and (f) utilized qualitative methodologies. Mixed age samples were included if (a) more than 50% of the sample was within the specified age range and (b) quotes could be directly linked to parents within the specified age range. Mixed aged samples were also included if less than 50% of the sample was within the age range if age related differences were discussed in the results and quotes could be directly linked to a parent within the specified age range. Mixed methods and observational studies containing qualitative data were included if they met the above inclusion criteria. Literature reviews, meta-analyses, chapters, psychometric studies, intervention studies, and theoretical or conceptual papers were excluded. As screening progressed, Google Scholar, Scopus, and hand searching were used to conduct forward and backward citation searches for additional articles which resulted in an additional 1699 articles. In total, 277 articles of the 1900 identified met inclusion or could not be excluded based on the content of the title and abstract alone. The full texts of these 277 articles were retrieved to assess for eligibility. If the first author was uncertain about inclusion of an article after full text review, she consulted with the second author to make the final decision.

### 2.3 Analysis

Meta-ethnography involves seven iterative phases. The first two phases are (1) identifying the focus of the review and synthesis, and (2) identifying and selecting what literature is relevant for inclusion (described above). The following five phases are (3) reading the studies to identify and extract initial concepts and themes, (4) rereading and engaging in constant comparison to determine how the studies' themes and concepts are related, (5) translating the studies into each other (e.g., exploring how concepts, themes, and metaphors extracted relate within and between studies), (6) synthesizing the translations into a line of argument by checking and cross checking the reviewer's interpretations across studies, and (7) expressing synthesis to target audience (Noblit and Hare, 1988).

This process was achieved by the first author initially reading each study and extracting basic methodological and contextual information during the full-text review phase. Next, she re-read all of the results and discussion sections from the included studies to refamiliarize herself with the data while engaging in reflexive memoing on (1) initial thoughts on identified themes and potential connections between papers and (2) reflections on how her identity may shape her interpretations of the data. She then imported all articles into ATLAS.ti 24.2.1 for Mac (ATLAS.ti Scientific Software Development GmbH, 2024) to extract and code first order constructs (e.g., direct quotes from caregivers and descriptions of behavior) in the results and discussion sections of each article totaling 413 quotes/descriptions. Initial codes used in this process were the second order constructs identified in each article (e.g., the themes that the quotes were used as exemplars of from the original manuscript). Identical themes were combined into single codes. She then began the process of arranging and re-arranging the data to develop groupings of similar themes. As a validity check, she discussed the findings and interpretations with the second and third authors (one with expertise in RES and one with expertise in early childhood development) throughout the process and re-read, recoded, and revised groupings, concepts, and interpretations based on these discussions. At this stage seven initial overarching groupings had emerged—(1) Parent's Experiences, (2) Parent's Reflections on the Process, (3) Parent's Values, Beliefs, and Intentions, (4) Methods of Communication, (5) Content/Message of Communication, (6) Child Traits, (7) Child (Racialized) Experiences. The first author then re-read each article and applied these larger grouping codes across all 413 quotes from the 27 articles. The translation process was further accomplished by examining patterns in co-occurrences in grouping codes and original theme codes within the data while re-reading and cross-checking the distribution of the of co-occurrences across studies to arrive at six final process focused themes. Lastly, the first author re-read each study one final time to confirm the presence or absence of the final themes derived from individual quotes in the authors' interpretations of their findings.

## 3 Results

**Figure 1:**
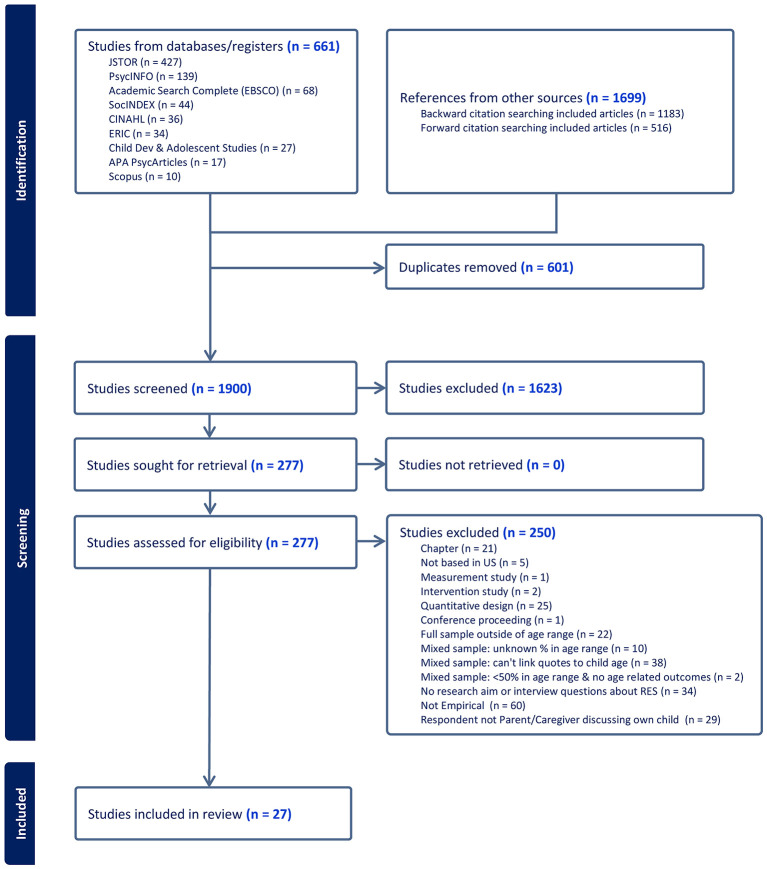
PRISMA flow diagram illustrating search and screening process.

Twenty-seven articles representing 23 studies met inclusion criteria with the remaining 250 records from the full-text review excluded for the following reasons: book chapter (*n* = 21), not an empirical article (e.g., review, opinion, essay, theoretical paper) (*n* = 60), intervention study (*n* = 2), psychometric study (*n* = 1), conference proceeding (*n* = 1), quantitative design (*n* = 25), not based in the US (*n* = 5), full sample outside of age range (*n* = 22), mixed sample with unknown percentage in age range (*n* = 10), mixed sample with less than 50% in age range and no age related outcomes (*n* = 2), mixed sample and didn't link quotes to child age (*n* = 38), no research aim or interview question about RES (*n* = 34), and respondent not parent/caregiver discussing own child (*n* = 29). One study utilizing mixed methods with a mixed-caregiver (e.g., familial and non-familial caregivers) sample was included as the authors reported on differences in racial socialization strategies between groups (Faragó, 2024). See Figure 1 for PRISMA figure.

Most studies were based on the accounts of Black parents raising Black children (*n* = 14). Latine parents raising Latine children (*n* = 5), Multiracial families (inclusive of transracial adoptees) (*n* = 2), and White parents raising White children (*n* = 2) were represented at lower rates. Four studies included mixed samples offering additional accounts from these groups with one study including accounts from Asian parents raising Asian children. Where reported, studies drew samples from the Southeast (*n* = 5), Mid-Atlantic (*n* = 4), Northeast (*n* = 4), Midwest (*n* = 3), Southwest (*n* = 2), and Westcoast (*n* = 2) regions of the US. The remaining studies sampled a broad US-based population and did not report the geographic breakdown of the samples. The included studies captured the experiences of approximately 714 caregivers of children ages 6 years and under. Parents' ages (where reported) range from 19 to 67. See Table 1 for study characteristics.

**Table 1:** Characteristics of included studies.

**References**	**Study aim**	**Design: methods | methodology**	**Participants**	**Geographic area**
(Aldoney and Cabrera 2016)	To explore how Latine immigrant parents instill bicultural values in their young children	Qualitative design: focus groups | Grounded theory	30 Immigrant Latine parents (20 mothers & 10 fathers) of children ages 2–4 enrolled in early head start	Washington, DC metro area
(Anderson et al. 2015)	To describe how minority parents help their young children navigate issues of race and racism and discuss implications this racial socialization may have for school readiness	Qualitative design: semi-structured focus groups split up by race/ethnicity and gender | Applied thematic analysis	60 African American, Latine, and Korean mothers & 54 fathers of a child under the age of 4 (*N* = 114)	Los Angeles, CA
(Bernard et al. 2023)	Examine how caregivers talk to their children about racism following incidents of anti-Black violence	Qualitative: semi-structured interviews | Inductive content analysis	12 Black mothers (ages 33–56) of children ages 5–29	Charleston, SC
(Blanchard et al. 2019)	Elucidate the ways in which African American parental childrearing perspectives and practices reflect attention on the salience of race and ethnicity	Qualitative: 3 semi-structured interviews per couple | Constructivist interpretive framework for phenomenological analysis and theoretical coding	6 African American married or cohabitating mother-father dyads (ages 25–43) raising male toddlers ages 16–33 mos	Southeastern county
(Calzada et al. 2010)	To investigate Latine parenting values (specifically *respeto*) and beliefs and socialization practices to derive a culturally informed model of Latine parenting	Qualitative: semi-structured focus groups | Constant comparative analysis	48 Dominican (*n* = 31; Mage = 35) and Mexican (*n* = 17; Mage = 31) female primary caregivers of children ages 3–6	Northeastern city
(Chaparro 2019)	To examine the intersections of race, language, and class in socialization within Two-Way Immersion programs	Qualitative: 18-months of participant observation, audio and video recording, informal and semi-structured interviews, and document collection | Discourse analytic ethnography	3 Multiracial (*n* = 1), White (*n* = 1), and Latine (*n* = 1) families with children in a bilingual kindergarten classroom	Philadelphia, PA
(Coard et al. 2004)	Explore the prevalence, rationale for use, content, methods, and modes of racial ethnic socialization strategies in low-income African American families	Qualitative: semi-structured interviews | Grounded theory	15 Low SES female African American primary caregivers (ages 25–67) to children ages 5–6	Northeastern city & Southeastern city
(Coates et al. 2024a)	To explore Black parents' rationale and reasons for utilization of specific ethnic-racial socialization strategies with their young children	Qualitative: focus-groups | Phenomenological reflexive thematic analysis	26 Black parents (ages 19–51) of children ages 1–5	Washington, DC metro area
(Coates et al. 2024b)	To understand whether, how, and to what extent Black parents engaged in preparation for bias with young children following the murders of George Floyd and other unarmed Black people during the pandemic	Qualitative: focus-groups | Reflexive thematic analysis	47 Black parents (ages 28–64) of children ages 2–10	Mid-Atlantic region
(Crowley and Curenton 2011)^a^	Exploring benefits of Mocha Moms and challenges among a group of African American mothers who voluntarily participated in a national organization and support group for mothers of color	Qualitative: semi-structured interviews | Modified grounded theory analysis	25 Black (*n* = 24) and Black-multiracial (*n* = 1) mothers (ages 26–44) of children ages 7 mo−14 years who are members of a national organization and support group for mothers of color	US-not otherwise specified
(Curenton et al. 2018)^a^	To examine middle-class, mostly married African American mothers' descriptions of mothering in relation to both the practical aspects and value-based aspects of child rearing	Qualitative: semi-structured interviews | Modified grounded theory analysis	25 Black (*n* = 24) and Black-multiracial (*n* = 1) mothers (ages 26–44) of children ages 7 mo−14 years who are members of a national organization and support group for mothers of color	US-not otherwise specified
(Delgado-Gaitan 1993)	To understand the principles by which immigrant and first-generation Mexican families raise their children	Qualitative: case-studies | Ethnography	10 immigrant (*n* = 5) and first generation (*n* = 5) Mexican families with a child 2–4 years old	Carpinteria, CA
(Doucet et al. 2018)	To explore how the intersection of parents' racism experiences and social class may play a role in race-related socialization during the early years	Qualitative: semi-structured interviews | Grounded theory	25 primary caregiving adults representing 13 African American middle-working class families (13 mothers, 7 fathers, 4 grandmothers, 1 great grandmother) with a child in preschool or kindergarten	Greensboro, NC
(Edwards and Few-Demo 2016)	To examine the maternal racial socialization of preschool-age children and capture dimensions of African American child development that are typically not accounted for by traditional child development theories	Qualitative: semi structured interview, child completed Clark Doll Test, then mother shown child's test and asked to reflect on how RES influenced results | Constructivist grounded theory	12 African American mothers (ages 23–36) of children ages 3–5	South Carolina
(Faragó 2024)	To explore racial socialization practices and dilemmas of caregivers nurturing BIPOC children	Mixed methods: open-ended online survey | Deductive thematic analysis	173 Caregivers (familial and non-familial) of BIPOC children ages 0–8	US-not otherwise specified
(Goldberg et al. 2016)	To examine adoptive parents' socialization practices regarding the minority statuses of their children (i.e., being of color, having two moms or dads)	Qualitative: in-depth interviews | Thematic analysis	Full sample: 82 parents in 41 couples Sub-sample for race-based questions: 33 lesbian (*n* = 11), gay male (*n* = 12) and heterosexual (*n* = 11) couples (ages 30–45) with children ages 4–11	US-not otherwise specified
(Heberle et al. 2021)	To understand White parents' aspirations and challenges in anti-racist parenting of young children	Qualitative: semi-structured interviews | Grounded theory	19 White parents (ages 29–44) of White children ages 3–6 who self-identified as antiracist	US-not otherwise specified
(Kim-Breunig and Vittrup 2024)	To explore the lived experiences of Asian-White interracial couples and their experiences raising their biracial children	Qualitative: semi-structured interviews | Phenomenology	10 Asian-White interracial married couples with children ages 3–31	US-not otherwise specified
(Leath et al. 2022)	To explore Black mothers' experiences of racial violence, racial grief, and coping & how these experiences inform childrearing practices	Qualitative: semi-structured interviews | Consensual qualitative methods	31 Black mothers of children ages 2 mo−21 years	US-not otherwise specified
(Lloyd 2022)	To explore racial socialization practices and goals among African American mothers of preschoolers	Mixed methods: survey & semi-structured interviews | Open & Axial coding	Full sample: 30 African American mothers of children ages 3–5. Qualitative sub-sample: 10 African American mothers with high & low RES scores	Omaha, NE
(Lloyd 2024)	To explore the complexity and role skin tone plays in Black parents' messages to their children	Mixed methods: survey & semi-structured interviews | Deductive thematic analysis	Full sample: 178 Black parents of children ages 5–8 Qualitative sub-sample: 10 Black parents who reported their child had light, dark, or medium skin tone	Nebraska
(Melzi et al. 2023)^b^	To understand immigrant Latine caregivers' experience of cultural negotiation in the context of parenting practices, child development, and schooling	Qualitative: focus-groups | Grounded theory	74 Latine (Immigrant *n* = 68) caregivers of preschool aged children	New York City, NY
(Njoroge et al. 2009)	To examine infants' awareness of racial differences and their parents' discussions about culture and race	Mixed methods: observation of child play & parent open ended survey | Constant comparative analysis	19 Infants and toddlers ages 6–36 months & their parents	Northeastern region
(Ochoa et al. 2023)^b^	To understand and compare salient parenting beliefs, attitudes, and self-reported practices as they pertained to supporting their children's development among both English- and Spanish-speaking caregivers	Qualitative: focus-groups | Grounded theory	112 Low-income Latine caregivers of preschool aged children	New York City, NY
(Pahlke et al. 2012)	To examine European American mothers' racial socialization of their 4–5-year-old children	Mixed methods: observation of dyad reading, mother & child questionnaires, mother interview | Modified analytic induction	84 White/European American mothers and their children ages 4–5	Texas
(Phelps and Sperry 2021)	To explore African American mothers' racial and academic socialization practices and beliefs that could influence early school experiences	Qualitative: semi-structured interviews & participant journaling | Phenomenology	11 African American mothers of children in kindergarten-3rd grade	Midwestern town
(Suizzo et al. 2008)	To clarify and describe three key domains of African American mothers' socialization of young children: racial socialization, academic socialization, and preparation for independence or interdependence	Qualitative: semi-structured interviews | Modified analytic induction	12 African American mothers of children ages 3–6	Southwestern region

^a, b^ indicate manuscripts from one larger study.

Through the extraction and translation process the following six themes were identified: (1) Parent Experiences Influence Their Values, Beliefs, and Intentions, (2) Parent Values, Beliefs, and Intentions—A Balancing Act, (3) RES Methods & Associated Messages, (4) Child Traits Influence the Process, (5) Child Racialized Experiences Influence the Process, and (6) Child Environment Influences the Process. Although these themes are conceptually related, they are treated as analytically distinct rather than nested, as they represent different dimensions of a multifaceted, non-linear process. Nesting would imply a linear progression from experience to belief to practice; however, the reviewed studies show that RES is non-linear, with beliefs not always enacted, practices sometimes conveying unintended messages, and parenting behaviors often shaped by distinct child and environmental factors. The following line of synthesis was developed from these concepts (each theme is described in more detail below): The process of racial-ethnic socialization for parents raising young children in the US is complex and multiply determined. Parents' experiences (both within, and for immigrant families in contrast to, the broader US social context) influence their cultural values, beliefs, and intentions to engage in specific parenting practices with their children. Parents often hold multiple beliefs and intentions at the same time, which can cause tension and require negotiation and balance between competing beliefs. Methods of RES and their associated messages are often dependent on parental intentions and cultural background. However, the RES process is not completely parent-driven. Child traits such as age and phenotype influence parents' behaviors, and especially for those who have chosen to wait to engage in RES, children's racialized experiences in environments outside of the parents' control often provide the catalyst for initiation of the process. See Table 2 for distribution of themes across studies.

**Table 2:** Distribution of themes across studies.

**References**	**Parent experiences influence their values beliefs and intentions**	**Parent values, beliefs, and intentions—A balancing act**	**RES methods & associated messages**	**Child traits influence the process**	**Child (racialized) experiences influence the process**	**Child environment influences the process**
(Aldoney and Cabrera 2016)	X	X	X	X		
(Anderson et al. 2015)	X	X	X		X	
(Bernard et al. 2023)	X	X	X	X	X	
(Blanchard et al. 2019)	X	X	X	X		X
(Calzada et al. 2010)	X	X	X	X		
(Chaparro 2019)	X	X	X	X		X
(Coard et al. 2004)	X	X	X	X	X	
(Coates et al. 2024a)	X	X	X	X		
(Coates et al. 2024b)	X	X	X	X	X	X
(Crowley and Curenton 2011)^a^	X	X	X	X	X	
(Curenton et al. 2018)^a^	X	X	X	X	X	X
(Delgado-Gaitan 1993)	X	X	X			
(Doucet et al. 2018)	X	X	X	X		X
(Edwards and Few-Demo 2016)		X	X	X	X	X
(Faragó 2024)	X	X	X	X	X	X
(Goldberg et al. 2016)	X	X	X	X	X	X
(Heberle et al. 2021)	X	X	X	X	X	X
(Kim-Breunig and Vittrup 2024)	X	X	X	X	X	X
(Leath et al. 2022)	X	X	X	X	X	X
(Lloyd 2022)	X	X	X	X	X	X
(Lloyd 2024)	X	X	X	X	X	X
(Melzi et al. 2023)^b^	X	X	X	X		X
(Njoroge et al. 2009)		X	X	X		
(Ochoa et al. 2023)^b^	X	X	X	X		X
(Pahlke et al. 2012)		X	X		X	
(Phelps and Sperry 2021)		X	X	X		X
(Suizzo et al. 2008)	X	X	X	X		X
Theme prevalence	85%	100%	100%	89%	55%	63%

^a, b^ indicate manuscripts from one larger study.

### 3.1 Theme 1: parent experiences influence their values, beliefs, and intentions

Across studies, both authors and parents described how parental life experiences and familial circumstance influenced the values, beliefs, and messages they intended to pass on to their own children as they relate to race, ethnicity, and culture (see Tables 2, 3 for distribution of themes and subthemes). Specifically, parents articulated how their own experiences—including their upbringing, encounters with racism, immigration histories, language proficiency, and class-based circumstances—influenced the cultural values they wished to pass down (Aldoney and Cabrera, 2016; Calzada et al., 2010; Coard et al., 2004; Coates et al., 2024a; Lloyd, 2022; Melzi et al., 2023; Ochoa et al., 2023; Suizzo et al., 2008), their intentions to prepare their children for encounters with racial bias (Bernard et al., 2023; Coard et al., 2004; Coates et al., 2024b; Doucet et al., 2018; Goldberg et al., 2016; Leath et al., 2022; Lloyd, 2022, 2024; Suizzo et al., 2008), optimistic egalitarian beliefs and views of the world (Anderson et al., 2015; Coard et al., 2004; Doucet et al., 2018), and antiracist ideologies (Heberle et al., 2021). Incorporation of parental experiences into their own RES practices has been referred to as “braided messaging” (Lloyd, 2022, 2024).

**Table 3:** Sub-themes of theme 1: parent experiences influence their values beliefs and intentions.

**Sub-theme**	**Articles**	**Prevalence (%)**
Own parents, family, and upbringing	Aldoney and Cabrera, 2016; Blanchard et al., 2019; Coates et al., 2024a; Curenton et al., 2018; Doucet et al., 2018; Edwards and Few-Demo, 2016; Heberle et al., 2021; Lloyd, 2022, 2024; Melzi et al., 2023; Ochoa et al., 2023; Suizzo et al., 2008	44
Racism/discrimination	Anderson et al., 2015; Bernard et al., 2023; Blanchard et al., 2019; Coard et al., 2004; Coates et al., 2024b; Crowley and Curenton, 2011; Doucet et al., 2018; Edwards and Few-Demo, 2016; Goldberg et al., 2016; Leath et al., 2022; Lloyd, 2022, 2024; Melzi et al., 2023	48
Immigration	Aldoney and Cabrera, 2016; Chaparro, 2019; Kim-Breunig and Vittrup, 2024; Melzi et al., 2023	15
Circumstance (i.e., parental intersectional identity, class, education, SES)	Aldoney and Cabrera, 2016; Coard et al., 2004; Curenton et al., 2018	11

For Black parents, personal experiences with racism, discrimination, and segregation were salient and explicit drivers of their RES beliefs and practices (Bernard et al., 2023; Blanchard et al., 2019; Coard et al., 2004; Coates et al., 2024b; Crowley and Curenton, 2011; Doucet et al., 2018; Edwards and Few-Demo, 2016; Leath et al., 2022; Lloyd, 2024). Some parents believed that the world was a kinder, less racialized place now than when they grew up—“race doesn't matter that much anymore. It's not like it was years ago” (Edwards and Few-Demo, 2016, p. 66).

However, for many parents of Black children (inclusive of Multiracial and adoptive families), the harsh reality of raising a child in a racist society was unavoidable due to vicarious trauma from witnessing highly publicized anti-black racial violence and killings (Bernard et al., 2023; Coates et al., 2024b; Faragó, 2024; Leath et al., 2022; Lloyd, 2022, 2024). The lament of a Black mother of a 4-year-old son and daughter captures this challenge:

It is traumatic to see Black bodies being abused and killed. It is traumatic. It shuts me down a little bit… Your kid might die for stealing a piece of candy, and that is something that White parents do not have to deal with at all (Leath et al., 2022, p. 3460).

Black parents indicated their own parents instilled values such as the importance of knowing history and the value of education that they wished to pass down to their own children (Phelps and Sperry, 2021; Suizzo et al., 2008). Many parents relayed how their parents communicated about race and racism, and how their childhood environments influenced their desires for their children (Blanchard et al., 2019; Coates et al., 2024a; Doucet et al., 2018; Lloyd, 2022, 2024). While some parents spoke of emulating their own parents' RES practices, some chose to parent their children in ways that went against their parents' practices. For example, a Black mother of a 2-year-old-son explained

I know for a good fact that speaking about race at a very young age affected me, because it made me perceive people differently, and I don't want that for my son (Coates et al., 2024a, p. 17).

Similarly, White parents' antiracist beliefs were often in reaction to their childhood color-mute or racist home environments—

I wanted to be more transparent with my kids about that stuff… in my household growing up like, if you said, if I had noticed like, ‘oh somebody is Black,' my parents would have been like, ‘shhh don't talk about that' (Heberle et al., 2021, p. 87).

For many Latine parents it was clear that “what was good about how our parents raised us, we will pass on to our children. What was bad, we will not pass on” (Calzada et al., 2010, p. 83). Across studies the “good” included cultural values such as respect (*respeto*), familism (*familismo*), and religious beliefs (Aldoney and Cabrera, 2016; Calzada et al., 2010; Melzi et al., 2023). The “bad” was the degree of strictness with which their parents enforced rules in the service of teaching *respeto* (Calzada et al., 2010), as well as traditional gender roles they viewed to be limiting for their children (Melzi et al., 2023; Ochoa et al., 2023).

Latine parents experienced challenges related to immigration such as separation from family, language barriers, and discrimination upon arrival to the United States (Aldoney and Cabrera, 2016; Melzi et al., 2023; Ochoa et al., 2023). However, parents used these challenges as a source of motivation in their parenting. For example, one parent said:

*Toda esa plenitud que nos hace falta al ver a nuestra familia, eso influye tal vez para ayudarnos a nosotros a ayudar a nuestros hijos* [the lack of completeness we feel as a result from being away from our family likely motivates us to help our children] (Melzi et al., 2023, p. 1418).

Another said “*Pero los hijos hacen que uno pierda el miedo que uno siente*. [But for our children we lose that fear that we feel]” in reference to the anxiety felt over their limited language proficiency in public spaces (Melzi et al., 2023, p. 1417). This reframing of parental hardship as motivational strength is similar to the theme of turning “pain into purpose” that Black parents voiced as a strategy to avoid parenting out of fear in a world where Black people are killed for “just existing” (Leath et al., 2022).

Physical distance and language barriers within families also motivated parents' beliefs about the importance of bilingualism (Kim-Breunig and Vittrup, 2024; Melzi et al., 2023; Ochoa et al., 2023). In the words of a Korean mother raising a 4-year-old biracial son:

Because they are all in Korea, I'm the only one here. So he does not get to spend time with my parents as much and when I do call them, he doesn't speak the language just yet. I do wonder if he spoke Korean then they would actually be able to have conversations (Kim-Breunig and Vittrup, 2024, pp. 20–21).

The intersection of parents' class and race/ethnicity on beliefs and intentions for their children was a prominent theme in Chaparro's (2019) ethnography of three students in a two-way bilingual immersion program. The impact of class and privilege was echoed throughout several other studies. (Doucet et al. 2018) found differences in Black parents' RES beliefs based on class (e.g., middle-class parents were more likely than working-class parents to draw connections between their experiences of racism and their RES practices). Parents with higher SES tended to have greater intentions to expose their children to diversity due to the environmental “challenge” of living in majority white neighborhoods (Blanchard et al., 2019; Crowley and Curenton, 2011; Heberle et al., 2021). In contrast, barriers associated with low-income and low levels of human capital (e.g., English proficiency and education) were a source of insecurity for Latine immigrant parents and negatively affected their belief in their abilities to help their children succeed. Irregular work hours affected their ability to establish routines and promote familism, and limited English proficiency affected their ability to assist with schoolwork which was associated with achievement related values (Aldoney and Cabrera, 2016).

### 3.2 Theme 2: parent values, beliefs, and intentions—A balancing act

Parents and caregivers in all 27 articles referenced their values, beliefs, and intentions for parenting behaviors associated with RES (see Tables 2, 4 for distribution of themes and subthemes). Intent for cultural socialization (e.g., specific cultural values and/or history, intent to instill racial-ethnic pride, or foster racial-ethnic awareness), preparation for bias socialization (e.g., to counter negative stereotypes and judgments), egalitarian socialization (e.g., beliefs that we're all created equal, race shouldn't matter and other qualities are more important), and beliefs about biculturalism and bilingualism were some of the most prominent topics. Parents also spoke of more general parenting beliefs and values such as aspirations for their children's success, safety, acceptance, and happiness. Most parents simultaneously expressed multiple beliefs, values, and intentions.

**Table 4:** Sub-themes of theme 2: parent values, beliefs, and intentions—A balancing act.

**Sub-theme**	**Articles**	**Prevalence (%)**
Egalitarian beliefs	Aldoney and Cabrera, 2016; Anderson et al., 2015; Coard et al., 2004; Coates et al., 2024a,b; Doucet et al., 2018; Edwards and Few-Demo, 2016; Faragó, 2024; Goldberg et al., 2016; Heberle et al., 2021; Kim-Breunig and Vittrup, 2024; Lloyd, 2022, 2024; Njoroge et al., 2009; Phelps and Sperry, 2021	55
Intention for preparation for bias	Anderson et al., 2015; Bernard et al., 2023; Coard et al., 2004; Coates et al., 2024b; Crowley and Curenton, 2011; Doucet et al., 2018; Goldberg et al., 2016; Heberle et al., 2021; Leath et al., 2022; Lloyd, 2022	37
Intention for cultural socialization	Aldoney and Cabrera, 2016; Bernard et al., 2023; Blanchard et al., 2019; Calzada et al., 2010; Chaparro, 2019; Coard et al., 2004; Coates et al., 2024a,b; Crowley and Curenton, 2011; Curenton et al., 2018; Delgado-Gaitan, 1993; Faragó, 2024; Goldberg et al., 2016; Heberle et al., 2021; Kim-Breunig and Vittrup, 2024; Lloyd, 2022, 2024; Melzi et al., 2023; Ochoa et al., 2023; Phelps and Sperry, 2021; Suizzo et al., 2008	78
Beliefs about bilingualism/biculturalism	Aldoney and Cabrera, 2016; Blanchard et al., 2019; Calzada et al., 2010; Chaparro, 2019; Coard et al., 2004; Delgado-Gaitan, 1993; Edwards and Few-Demo, 2016; Kim-Breunig and Vittrup, 2024; Melzi et al., 2023; Ochoa et al., 2023	37
Broad parenting beliefs/intentions (child safety, success, happiness)	Aldoney and Cabrera, 2016; Anderson et al., 2015; Bernard et al., 2023; Blanchard et al., 2019; Chaparro, 2019; Coard et al., 2004; Coates et al., 2024a; Crowley and Curenton, 2011; Curenton et al., 2018; Doucet et al., 2018; Edwards and Few-Demo, 2016; Goldberg et al., 2016; Heberle et al., 2021; Kim-Breunig and Vittrup, 2024; Leath et al., 2022; Lloyd, 2022, 2024; Melzi et al., 2023; Ochoa et al., 2023; Phelps and Sperry, 2021; Suizzo et al., 2008	78
Tension or balance in competing beliefs	Aldoney and Cabrera, 2016; Anderson et al., 2015; Calzada et al., 2010; Coard et al., 2004; Delgado-Gaitan, 1993; Faragó, 2024; Heberle et al., 2021; Ochoa et al., 2023; Suizzo et al., 2008	33

In some cases, parents' beliefs were congruent and led to clear messaging. For example, Black parents communicated messages about racial excellence and achievement to their children as a means to ensure their children's future success and to prepare them for future encounters with racial discrimination and bias that may limit success (Coard et al., 2004; Doucet et al., 2018; Suizzo et al., 2008). As one mother shared,

I think he needs to know that if he is smarter, that they may not necessarily acknowledge it. I want him to know that he does have to work harder to prove himself, but I want him to work hard (Doucet et al., 2018, p. 74).

In other cases, there was tension and even conflict in parents' beliefs and intentions. This tension was more frequently identified by the authors than the parents themselves. For instance, (Anderson et al. 2015) found that parents faced many experiences with- and had keen awareness of- the pervasiveness of institutional racism (e.g., “just because you're black, you're profiled”). Parents vocalized understanding the need for their children to eventually understand their racial-ethnic identity so that “when they face racial discrimination later on, they [don't] go through confusion” (Anderson et al., 2015, p. 4).

However, almost all parents in their sample still espoused overwhelmingly egalitarian beliefs when it came to raising their children such that “success has nothing to do with race” or “the child himself or herself is the most important thing” (Anderson et al., 2015, p. 4). Similarly, (Heberle et al. 2021) found that despite their sample of White parents identifying as anti-racist, their parenting practices mostly focused on helping their children identify and speak out against interpersonal racism. Parents did very little to address or resist the systemic privileges afforded to them through Whiteness.

Authors in three studies identified parents' ability to find balance in tensions between racial-ethnic/cultural values and mainstream values to ensure their children's future success (Aldoney and Cabrera, 2016; Delgado-Gaitan, 1993; Suizzo et al., 2008). Both Black and Latine parents were able to find balance between values of independence and interdependence. Black parents spoke of *both* as important cultural values to pass down to their children and did not present them as conflictual. As a Black mother of a 5-year-old-boy explained,

family [is important]… to let him know that I have his back, not so much that I'm trying to influence you, but I am trying to influence you in that I'm trying to get you to be your own person (Suizzo et al., 2008, p. 304).

As Latine families gained more experience in the US (e.g., both within generations and across generations) parents encouraged more independence in their children such as “you need to learn how to do it yourself” (Delgado-Gaitan, 1993, p. 418). In both Black and Latine families, values of independence were not viewed to be at odds with the interdependence value of family unity, and independence was still situated within interdependence such that independent behaviors were viewed as direct reflections of their families and communities (Calzada et al., 2010; Coard et al., 2004; Delgado-Gaitan, 1993). (Aldoney and Cabrera 2016) also identified that holding both “new” beliefs and heritage cultural beliefs was possible so long as the two were not viewed to be at odds. For instance, immigrant families did not see *respeto* to be at odds with the American cultural expectation/value of critical thinking and open communication (Calzada et al., 2010; Delgado-Gaitan, 1993). Such adaptations of beliefs and values were often to ensure their children's future success in the United States and reflected a bicultural orientation toward parenting. Even Latine parents with less bicultural parenting beliefs had a strong desire for bilingualism in their children. As one parent reflected,

It's good to learn two different languages for the future, jobs, anything. Like I said, a person doesn't speak English, you know how to adapt and speak Spanish. So, I like that he has that advantage (Ochoa et al., 2023, p. 4005).

While parental awareness was less common, parents were not always blind to the tension and conflict in their beliefs. For example, White caregivers of minoritized and White children were concerned over the potential impacts that their own unaddressed biases and blind spots might have on their children (Faragó, 2024; Heberle et al., 2021). *One* parent lamented,

… I've got a lot of history of blindness myself. And so I worry… is the thing I'm saying to them counterproductive actually? Like am I actually enforcing narratives that are racist? (Heberle et al., 2021, p. 90).

### 3.3 Theme 3: racial-ethnic socialization methods and associated messages

Parents and caregivers across all 27 articles referenced methods of RES such as oral communication, exposure to diversity and/or own culture, and behavioral methods (see Table 5 for methods) with explicit or implicit (e.g., author-classified) connections to the content and underlying messages of those methods.

**Table 5:** Methods of RES.

**Method**	**Articles**	**Prevalence (%)**
Avoidance	Anderson et al., 2015; Coates et al., 2024a,b; Edwards and Few-Demo, 2016; Goldberg et al., 2016; Heberle et al., 2021; Kim-Breunig and Vittrup, 2024; Leath et al., 2022; Lloyd, 2024; Njoroge et al., 2009; Pahlke et al., 2012; Phelps and Sperry, 2021; Suizzo et al., 2008	48
Diversity exposure	Blanchard et al., 2019; Chaparro, 2019; Coard et al., 2004; Coates et al., 2024a; Edwards and Few-Demo, 2016; Faragó, 2024; Heberle et al., 2021; Njoroge et al., 2009; Pahlke et al., 2012	33
Exposure to heritage people/culture	Aldoney and Cabrera, 2016; Blanchard et al., 2019; Chaparro, 2019; Coard et al., 2004; Coates et al., 2024a; Crowley and Curenton, 2011; Delgado-Gaitan, 1993; Faragó, 2024; Goldberg et al., 2016; Kim-Breunig and Vittrup, 2024; Lloyd, 2022, 2024; Ochoa et al., 2023; Suizzo et al., 2008	51
Language exposure	Calzada et al., 2010; Chaparro, 2019; Goldberg et al., 2016; Kim-Breunig and Vittrup, 2024; Melzi et al., 2023; Ochoa et al., 2023	22
Manage/correct behavior	Bernard et al., 2023; Blanchard et al., 2019; Calzada et al., 2010; Coard et al., 2004; Delgado-Gaitan, 1993; Edwards and Few-Demo, 2016; Lloyd, 2022, 2024; Melzi et al., 2023; Ochoa et al., 2023	37
Modeling	Aldoney and Cabrera, 2016; Blanchard et al., 2019; Calzada et al., 2010; Coard et al., 2004; Curenton et al., 2018; Delgado-Gaitan, 1993; Edwards and Few-Demo, 2016; Heberle et al., 2021; Leath et al., 2022; Melzi et al., 2023; Ochoa et al., 2023	41
Oral (teaching/advice)	Aldoney and Cabrera, 2016; Anderson et al., 2015; Bernard et al., 2023; Blanchard et al., 2019; Calzada et al., 2010; Coard et al., 2004; Coates et al., 2024a,b; Doucet et al., 2018; Edwards and Few-Demo, 2016; Faragó, 2024; Goldberg et al., 2016; Heberle et al., 2021; Kim-Breunig and Vittrup, 2024; Leath et al., 2022; Lloyd, 2022, 2024; Melzi et al., 2023; Ochoa et al., 2023; Pahlke et al., 2012	74
Oral via books, movies, TV, photos	Coard et al., 2004; Coates et al., 2024a,b; Edwards and Few-Demo, 2016; Faragó, 2024; Goldberg et al., 2016; Heberle et al., 2021; Lloyd, 2024; Pahlke et al., 2012	33
Routines	Aldoney and Cabrera, 2016; Blanchard et al., 2019; Coates et al., 2024a; Kim-Breunig and Vittrup, 2024; Lloyd, 2022, 2024; Ochoa et al., 2023; Suizzo et al., 2008	30

One of the most widely used methods across all racial-ethnic groups was oral communication. Latine parents specifically reported giving advice (*consejo*) to their children. In the words of a Latino father of a preschool-aged girl, “… talking to them since they are little kids, that is the most important… then they start developing a sense of what is right and what is not” (Aldoney and Cabrera, 2016, p. 3612). Similarly, Black parents and parents in Multiracial families reported explicit “teaching” conversations with their children about history, “the struggle,” and culture (Coard et al., 2004; Coates et al., 2024a; Edwards and Few-Demo, 2016; Goldberg et al., 2016; Phelps and Sperry, 2021; Suizzo et al., 2008). In the words of one Black mother, “you can't know where you are going if you don't know where you came from” (Coard et al., 2004, p. 286).

Parents across all race-ethnicities report using books, TV, photos, and movies as catalysts for conversations about race and culture (Coard et al., 2004; Coates et al., 2024a,b; Edwards and Few-Demo, 2016; Faragó, 2024; Goldberg et al., 2016; Heberle et al., 2021; Lloyd, 2024). Some parents reported confidently and successfully using these methods—“I began weaving racial literacy into my children's lives… [we] read the book ‘Antiracist Baby' to introduce the term ‘antiracism”' (Faragó, 2024, p. 8). However, not all parents felt prepared for discussions the materials prompted. As one Black mother described, “And so that was kind of a teachable moment, but I didn't know exactly what to say to him” (Lloyd, 2024, p. 1596). (Pahlke et al. 2012) found that despite the majority of White mothers having pro-black attitudes and intentions to engage in RES with their children, when observed reading two picture books expected to elicit discussion about race with their children, they made very few explicit comments about race, and rarely challenged or contradicted their children's racist or biased comments.

Behavioral methods were also prevalent across racial-ethnic groups, with parents utilizing RES methods such as exposure (to heritage culture, language, and diverse environments), modeling, maintaining routines, managing and correcting their children's behavior, and active avoidance of conversations around race, ethnicity, and culture. Avoidance behaviors included redirecting or deflecting in conversations about skin-tone and race when brought up by children. A white father recalled a conversation he had with his 6-year-old biracial daughter, “So yesterday I half-jokingly said, ‘That Black man is wearing blue shoes. Why don't you talk about the man in the blue shoes [instead of his race]?”' (Goldberg et al., 2016, p. 283).

Parents reported intentionally modeling behaviors and language they hoped their children would see and emulate, and also felt maintaining routines was an important aspect of parenting linked with RES (Aldoney and Cabrera, 2016; Blanchard et al., 2019; Coard et al., 2004; Coates et al., 2024a; Curenton et al., 2018; Delgado-Gaitan, 1993; Edwards and Few-Demo, 2016; Heberle et al., 2021; Kim-Breunig and Vittrup, 2024; Leath et al., 2022; Lloyd, 2022, 2024; Melzi et al., 2023; Ochoa et al., 2023; Suizzo et al., 2008). Routines included activities such as mealtimes, prayers, chores, and homework. Black parents spoke about grooming routines where they provided their children with affirmations and modeled positive language about their hair. A Black mother of a 3-year-old Black girl shared,

… when I'm combing [her] hair, and there might be kinks in it or something… I just never tried to use negative words… I just say… your hair is strong… And you can do all kinds of things with your hair (Lloyd, 2022, p. 1870).

One Latina mother shared the importance of modeling good study habits with a regular study routine she shares with her preschool-aged children,

with them, well I do my homework on the weekends and I give them a book that they read [or] painting and as they see me learning English… it also helps (Aldoney and Cabrera, 2016, p. 3612).

The content or message of some behavioral methods was clear to authors and congruent with the method (e.g., cultural socialization messages from exposure to one's own race, ethnicity, or culture, and egalitarian or color-blind messages from avoidance; Anderson et al., 2015; Blanchard et al., 2019; Coard et al., 2004; Coates et al., 2024b; Faragó, 2024; Goldberg et al., 2016; Lloyd, 2024; Ochoa et al., 2023; Suizzo et al., 2008). However, the messages conveyed by many of these methods varied by the race-ethnicity and intentions and beliefs of the parent. For example, when White parents of White children exposed their children to diversity it communicated color-conscious messages (Heberle et al., 2021). However, for parents of minoritized children encouragement of diverse friendships and exposure to diverse groups may communicate an egalitarian message or be indicative of adaptive socialization efforts within the broader category of preparation for bias (Coard et al., 2004; Edwards and Few-Demo, 2016). This is captured in a Black couple's exchange about their efforts to imbue the ability to code switch in their son via exposure to diverse environments:

Father: … They have to know some kids from this side of the street and that side… a little bit of everybody.

Mother: … [and] that they can change their talk depending on the environment, but they don't have to change who they are (Blanchard et al., 2019, p. 395).

Black and Latine parents both reported managing and correcting their children's behavior as a method of RES; however, the associated messages varied by race-ethnicity. Latine families' intended message is one of cultural socialization (i.e., *respeto*). Black families managed behavior to communicate preparation for bias (e.g., ensuring behavior that counteracts stereotypes; Bernard et al., 2023; Calzada et al., 2010; Delgado-Gaitan, 1993; Edwards and Few-Demo, 2016; Lloyd, 2022; Ochoa et al., 2023). A Black mother of a preschool-aged son shared,

it's just sad because I have to… tell my kids how to carry themselves… I have to sit here and be like… because you're hanging around White people, you've got to sit here and act like you're proper and stuff like that (Lloyd, 2022, p. 1869).

In contrast, a Latine parent of a preschool-aged son described how she teaches her son

to respect their teachers, older adults, and even older siblings too… sometimes my son tells me ‘tú' [informal you] and I tell him don't use ‘tú' use ‘usted' [formal you] (Ochoa et al., 2023, p. 4008).

### 3.4 Theme 4: child traits influence the process

Caregivers in 23 of the 27 articles made decisions about RES based on traits of their children such as age/developmental level, gender, phenotype, and gender/phenotype match with their parents (see Tables 2, 6 for distribution of themes and subthemes). This theme also highlights how these traits guide the timing, content, and focus of RES practices.

**Table 6:** Subthemes of theme 4: child traits influence the process.

**Sub-theme**	**Articles**	**Prevalence (%)**
Age/developmental considerations	Bernard et al., 2023; Blanchard et al., 2019; Calzada et al., 2010; Coates et al., 2024a,b; Doucet et al., 2018; Edwards and Few-Demo, 2016; Faragó, 2024; Goldberg et al., 2016; Heberle et al., 2021; Kim-Breunig and Vittrup, 2024; Leath et al., 2022; Lloyd, 2022, 2024; Njoroge et al., 2009; Suizzo et al., 2008	59
Gender	Blanchard et al., 2019; Coard et al., 2004; Coates et al., 2024a; Crowley and Curenton, 2011; Curenton et al., 2018; Edwards and Few-Demo, 2016; Lloyd, 2022, 2024; Melzi et al., 2023; Ochoa et al., 2023; Phelps and Sperry, 2021; Suizzo et al., 2008	44
Phenotype	Chaparro, 2019; Doucet et al., 2018; Kim-Breunig and Vittrup, 2024; Lloyd, 2024	15
Match to caregivers	Curenton et al., 2018; Edwards and Few-Demo, 2016; Goldberg et al., 2016; Lloyd, 2024; Melzi et al., 2023; Ochoa et al., 2023	22

Parents most frequently cited their children's age or developmental level as major determinants of their RES practices. Latina mothers based their methods of teaching and expectations for their children on their child's age. Mothers were in agreement that 3-year-olds may have difficulty acting with *respeto*, but “at 4 years old, [they are] a person that understands what is right and what is wrong” (Calzada et al., 2010, p. 83).

Black parents and parents in Multiracial families felt they should tailor messages about history to be age appropriate by avoiding discussions of harsh or negative events (Goldberg et al., 2016; Suizzo et al., 2008). One Black mother of a Black 5-year-old girl explained:

… just a lot of the history and… encouragement of the things that they went through, but not so much the negative. You know, wonderful reminders and just say these are the beautiful things, but you know what, there will always be a hater (Suizzo et al., 2008, p. 299).

Similarly, parents tended to focus their initial RES efforts on naming differences and instilling pride and confidence in their children as a way to tailor their messages to their children's developmental capacities (Bernard et al., 2023; Faragó, 2024; Goldberg et al., 2016). In the words of a Black mother explaining how age played a role in her approach with her daughters ages six, 12, and 18:

So at that early age the conversation [is] basically like, “Yeah, you know, you are different. People have different skin tones.” … but as the kids grow older… we talked about seeing [things] on TV and experiencing things in the classroom, it gets a little harsher (Bernard et al., 2023, p. 43).

Often these decisions were driven by fear of the negative consequences of engaging in preparation for bias too young which was evident in parents' intent to “keep [their] innocence” (Coates et al., 2024b, p. 1732). There was a similar belief amongst other parents that their children were too young to understand the concepts of race and racism. For instance, a mother of a toddler stated:

I don't think he knows anything about “race” or skin color… if you ask him what his “race” is he probably won't understand the question, but if you ask him what his skin color is, I think he will say “blue” just because that's his favorite color, and he doesn't understand (Njoroge et al., 2009, p. 562).

For the parents who believed their children were too young to understand racialized concepts, conversations relating to preparation for bias would be damaging, or explicit RES was unnecessary for their child, the most commonly cited tactic was to wait with the attitude of “when he's ready, I'll be ready” (Edwards and Few-Demo, 2016, p. 62). However, parents rarely identified what an indicator of readiness might be other than their child asking questions or encountering a “situation” that necessitated discussion. In the words of a Black mother of a 3-year-old Black daughter,

When? I can't pinpoint when. I don't know. I mean, tomorrow a situation could happen, we gotta go that route. I don't know, kindergarten, first grade. I really don't know (Suizzo et al., 2008, p. 299).

Children's racial and gender phenotype influenced parents' RES intentions and practices. Parents of Black boys and girls were particularly concerned about countering negative stereotypes of their children. Parents of Black boys voiced concern over their children's safety—especially with respect to potential encounters with police (Coard et al., 2004; Crowley and Curenton, 2011; Curenton et al., 2018; Lloyd, 2022, 2024). In the words of one mother of an African American son,

… African American boys are either getting arrested or getting killed at an alarming rate, and so your concern is: What do I need to do to protect my child? (Crowley and Curenton, 2011, p. 7)

In contrast, mothers of Black girls voiced concerns over instilling pride in the face of European beauty standards (Coates et al., 2024a; Phelps and Sperry, 2021; Lloyd, 2022). In the words of an African American mother of a 6-year-old girl,

it's [being Brown] not a disadvantage. You're special… making sure that they know that they're beautiful and that's why people are trying to take what they got (Coates et al., 2024b, p. 1734).

With respect to gender, Latine parents also made efforts to empower their daughters to pursue careers and to encourage their sons to take responsibility for household chores in efforts to promote egalitarian gender roles (Melzi et al., 2023; Ochoa et al., 2023).

(Lloyd 2024) found differences in Black parents' descriptions of their RES practices based on their children's skin-tone. For instance, parents of Black children with light skin-tone tended to avoid discussions of race related topics with their children as they believed that their children “may have a little more success in life just because [they're] a lighter skin-tone” (Lloyd, 2024, p. 1596).

Similarly, the parents of a Costa-Rican-White Biracial kindergartener referenced their son's appearance being “not a white boy with blue eyes and blonde hair” as a reason they wanted their son to “develop his [Biracial] Spanish identity” (Chaparro, 2019, p. 5). Another father of an Asian-White Biracial preschool-aged girl raised concerns over his daughter's exposure to fetishizing comments—

All these sayings, ‘Oh, mixed-race children are better looking than normal children.' On one hand, it's a nice thing but I don't want her to internalize that too much (Kim-Breunig and Vittrup, 2024, p. 21).

Children's match to parents' and caregivers' race, skin-tone and gender also appear to be salient determinants of the RES process. White adoptive parents of Biracial children with lighter skin-tones either avoided conversations about race altogether because their child “looks like us,” or deflected in conversations where their child pointed out race (Goldberg et al., 2016). For several interracial couples raising Biracial children, the task of RES was largely undertaken by the parent who shared minoritized status with the child (Goldberg et al., 2016; Kim-Breunig and Vittrup, 2024). Gender match was noted as important for boys in relation to modeling RES methods with Latine mothers encouraging their husbands to model “50/50” split of household chores (Melzi et al., 2023; Ochoa et al., 2023). Black mothers raising Black sons also highlighted the importance of positive male role models in addition to “nurture them [sons] more to raise them in the right direction [if] they don't have fathers around” (Curenton et al., 2018; Edwards and Few-Demo, 2016, p. 62; Lloyd, 2024).

### 3.5 Theme 5: child (racialized) experiences influence the process

In 15 of the 27 articles parents across all racial-ethnic groups reported that their child had already been exposed to racialized concepts or experienced racism/discrimination (see Tables 2, 7 for distribution of themes and sub-themes). Across articles parents and authors highlighted the varied ways young children encountered racism and racialized concepts (both directly and inadvertently) and how parents across racial-ethnic groups responded to these exposures with a mixture of concern, intervention, and uncertainty.

**Table 7:** Subthemes of theme 5: child (racialized) experiences influence the process.

**Sub-theme**	**Articles**	**Prevalence (%)**
Child makes racist remark	Faragó, 2024; Heberle et al., 2021; Pahlke et al., 2012	11
Direct racism/discrimination	Anderson et al., 2015; Bernard et al., 2023; Crowley and Curenton, 2011; Curenton et al., 2018; Faragó, 2024; Goldberg et al., 2016; Leath et al., 2022	26
Inadvertent exposure to racism or racial concepts	Anderson et al., 2015; Coard et al., 2004; Coates et al., 2024b; Edwards and Few-Demo, 2016; Heberle et al., 2021; Kim-Breunig and Vittrup, 2024; Leath et al., 2022; Lloyd, 2022, 2024	33

Parents recounted stories of their children's exposure to racist comments—“And to teach another 4-year-old child racial slurs!?” (Bernard et al., 2023, p. 42)—and/or microaggressions such as people asking the parent of a Biracial child “oh, well, what is she?” (Anderson et al., 2015, p. 4). Alarmingly, parents reported that their pre-school and kindergarten aged children were already experiencing discrimination via social exclusion and racially motivated bullying at school (Anderson et al., 2015; Crowley and Curenton, 2011; Curenton et al., 2018; Edwards and Few-Demo, 2016). In such cases, parents intervened with teachers and parents of other children involved, but voiced feeling “heartbroken” due to the parents of the perpetrating children not taking responsibility or insisting they “didn't know where that [racist behavior] came from” (Crowley and Curenton, 2011, p. 8; Curenton et al., 2018).

While less common, some parents admitted that their own children had made racist comments, leaving them feeling anxious and unsure of how to respond (Faragó, 2024; Heberle et al., 2021).

Parents also reported that their children frequently had inadvertent exposures to the concept of discrimination or racism (e.g., brought up by a teacher, overheard adults talking, saw news stories about racial violence, or witnessed police profiling in their neighborhood)—especially in the wake of highly publicized instances of racial violence (Coates et al., 2024b; Edwards and Few-Demo, 2016; Lloyd, 2024). Parents' responses to inadvertent exposures ranged from “having a very limited discussion” (Kim-Breunig and Vittrup, 2024, p. 16) to modeling- or allowing their children to participate in social justice activities such as Black Lives Matter marches (Faragó, 2024; Leath et al., 2022; Lloyd, 2024). A common sentiment amongst parents whose children had an inadvertent exposure was a sense of unpreparedness. A Black mother of a kindergartner described, “it came up in kindergarten from a teacher, and they spent like 8 weeks in class discussing it… I was very surprised and not prepared to follow up” (Coates et al., 2024b, p. 1734).

Other parents questioned whether or not they were effective in communicating the right message in the moment (Coates et al., 2024b; Heberle et al., 2021; Leath et al., 2022; Lloyd, 2024).

### 3.6 Theme 6: child environment influences the process

Parents and authors in 17 articles (see Table 2) spoke about how children's environments, shaped their racialized experiences and language exposure. These environments were often outside of the parents' control leading parents across groups to respond through proactive socialization strategies, environmental shaping, and reliance on broader networks.

While some of the stories of children's first encounters with racism and discrimination happened at home (e.g., overhearing adults and the news), many happened in environments outside parents' control such as school, their neighborhoods, or with other family members. Some parents voiced understanding the inevitability of their children's racialized experiences outside of their realm of influence, which left them feeling powerless over controlling messages their children receive. However, many Black parents engaged in preparation for bias and cultural socialization tactics as a means to pre-emptively counteract external influences (Coates et al., 2024a,b; Lloyd, 2022, 2024). In the words of one Black parent,

Sometimes I feel bad because it's like I can't be there for everything. I can't fight all their battles. But I feel like I have to make them strong enough to be able to deal with it. And be proud of it and continue on (Coard et al., 2004, p. 284).

A few Black parents attempted to proactively intervene in their children's environments by talking to teachers and other adults in their children's lives to ensure their children were receiving messages about race that aligned with their beliefs (Crowley and Curenton, 2011; Edwards and Few-Demo, 2016; Suizzo et al., 2008). For example, one Black mother tried “to do a lot of front-end conversation with teachers of how they discipline” and another tried “to encourage family and friends, if they're going to buy a doll, that she be African American” (Lloyd, 2022; Suizzo et al., 2008, p. 304).

In pursuit of Latine parents' bilingual socialization goals, parents who spoke little to no Spanish often relied on extended family to provide heritage language exposure to their children “because who is going to teach the kids Spanish?” (Ochoa et al., 2023, p. 4006). Parents who spoke little to no English relied on school and other relatives to provide English language exposure (Delgado-Gaitan, 1993; Melzi et al., 2023; Ochoa et al., 2023). This sometimes took the form of interactions between siblings. One mother described how her 6-year-old daughter's play helped her son develop English language skills,

… There are days when she pretends to be a teacher… and says: ‘Kevin sit down and pay attention to me.' She writes his name, writes her name and tells him: you are the student and I'm the teacher (Melzi et al., 2023, p. 1420).

Several parents provided egalitarian socialization messages due to concerns over their children feeling singled-out in predominantly white school environments (Lloyd, 2024; Phelps and Sperry, 2021). Others made decisions about what school to send their children to, what neighborhoods to live in, and what extracurricular activities to enroll their children in with exposure goals in mind (e.g., to diversity, same race-ethnicity others, and language; Blanchard et al., 2019; Heberle et al., 2021; Lloyd, 2022, 2024).

## 4 Discussion

This review of the literature on parental RES aimed to uncover information about the process of RES during early childhood that has not been captured in current reviews and quantitative studies. Results of analysis of 27 studies suggest that parental RES during early childhood is a dynamic, contextually influenced, and developmentally sensitive process. The early childhood RES process emerged through six interrelated themes: (1) Parent Experiences Influence Their Values, Beliefs, and Intentions, (2) Parent Values, Beliefs, and Intentions—A Balancing Act, (3) RES Methods & Associated Messages, (4) Child Traits Influence the Process, (5) Child Racialized Experiences Influence the Process, and (6) Child Environment Influences the Process.

The first theme highlighted how parents' experiences (e.g., their own socialization, experiences with racism/discrimination, immigration, and familial context) influenced their intentions, beliefs, and values about RES practices with their children. This aligns with Yasui's (2015) PMERS model of RES which highlights how social experiences and past behaviors influence intentions for RES via implicit and explicit attitudes and beliefs about behavior, race, social norms, and agency. For Black parents specifically, repeated exposure to racial violence had varied effects. Some felt immobilized, while for others it motivated desire to engage in activism (Leath et al., 2022). Similar patterns may emerge in other minoritized communities, particularly among Latine immigrant families, who are facing similar vicarious exposure and increased probability of experiencing personal effects of structural and systemic racism and violence. It may be especially important to encourage familial communication about salient identity-related threats being publicized in the news, as avoidant family communication styles have been associated with negative child mental health outcomes during times of ongoing threat or crisis (Malloy et al., 2024). Media exposure to racial violence was also present in recent reports of parents from other racial-ethnic groups (Faragó, 2024; Heberle et al., 2021; Kim-Breunig and Vittrup, 2024). These parents not only expressed worry and fear for their children but also noted that discussions about race had become unavoidable. Given the current political climate—characterized by opposition to diversity, equity, and inclusion (DEI) initiatives, anti-discrimination laws, and immigration protections—it is essential to continue to examine how parents respond to highly publicized acts of violence and racism and how they integrate these events into their parenting.

The second theme further explored parents' values, beliefs, and intentions for RES. Parents often held multiple beliefs simultaneously—some congruent and clearly reflected in their RES practices, and others in tension with one another. Although some of their competing beliefs were evidenced via ambivalent behavior or expression of broad beliefs about children that were incongruent with other expressions of their own personal parenting beliefs, there were examples of families negotiating “bicultural parenting” whereby they were able to hold two traditionally “opposing” cultural values/beliefs at the same time. The evidence of “bicultural parenting” observed in this review illustrates that these tensions may reflect not only competing RES goals but also parents' navigation of dual cultural value systems, consistent with integrative acculturation approaches (Boski, 2008).

The third theme highlighted the variety of methods and associated RES messages parents utilize during the early childhood period. Although parents endorsed using oral communication methods of RES, they also reported using behavioral methods such as modeling, maintaining daily routines, and even correcting their children's behavior. These findings align with evidence of behavioral socialization approaches during early childhood in the broader socialization literature (Zinsser et al., 2021). Family practices such as mealtimes, grooming, and discipline were only captured as RES in the current review if the parent consciously imbued these routines with RES intentions, if the interview questions pulled for these types of descriptions, or if the author classified these routines as RES. Despite being commonly used techniques, these daily routines and practices are not currently captured in most quantitative studies of RES. Further examination of daily routines mentioned by parents in this review may not necessitate conducting new studies, as existing naturalistic observational studies of families such as mealtime studies and parent-child language interaction studies likely capture RES within their observations. Coding schemes have been developed to classify verbal messages that could easily be applied to these data (Aguayo et al., 2021), and analytic techniques such as structural topic modeling (Roberts et al., 2014) could also be used to more quickly code large amounts of secondary data and identify patterns (e.g., tone, emotional affect) in verbal messages associated with specific types of daily routines.

Use of behavioral methods also highlights the importance of parental intention and the alignment between the messages parents intend to communicate and those children receive through routine or behavioral practices. Across studies, Black and Latine parents used behavior management or correction as RES strategies, though with different socialization goals (e.g., preparation for bias among Black families and cultural socialization among Latine families). Yet there is no evidence confirming these practices communicate the intended messages, and research with older youth indicates that parents' intended RES messages often diverge from what children perceive (Hughes et al., 2009; Thomas and King, 2007). This discrepancy highlights the limitations of relying on parental report to assess the messages youth receive. Concordance would be difficult to study through traditional methods (e.g., comparison of parent and child interviews) during early childhood. However, retrospective dyadic interviews could offer unique perspectives on the alignment between the messages parents intend to communicate and how children understand them.

The fourth theme elucidated various child traits that influenced parents' RES intentions and practices. Parents most frequently cited their child's age or developmental level as a major consideration when approaching or avoiding RES with their children. Parents' avoidance of racial topics and judgment of their children's developmental capacities align with the (Sullivan et al. 2021)'s findings that most adults misjudge the ages at which children achieve race-related developmental milestones. Children's racial phenotype also emerged as a salient determinant of parents' RES practices. There is preliminary evidence of this phenomena in African American families and Multiracial families where colorism and familial racism appear to be important yet understudied constructs (Christophe et al., 2024; Wilder and Cain, 2011). A small number of studies also highlighted how intersectional gender and phenotype match/mismatch to parents contributes to RES intentions and practices. This was especially salient for families with male Black and Latino children, and families with multiracial children.

The fifth theme highlighted that children across studies were introduced to racialized concepts or experienced direct acts of racism and discrimination at a very early age. This is supported by extant research showing that children's prejudice toward outgroups steadily rises during the preschool years and peaks by age seven (Raabe and Beelmann, 2011). Many models of parental RES are unidirectional without consideration of how children's experiences influence the process of RES. However, (Williams et al. 2020b)'s lifespan model of ethnic-racial identity highlights that experiences of discrimination in addition to RES are identity relevant experiences and can happen across the lifespan. When faced with their children's racialized experiences, parents in this review often felt unprepared, uncertain of what to say, or questioned their decisions regarding RES (Coates et al., 2024b; Faragó, 2024; Heberle et al., 2021; Leath et al., 2022; Lloyd, 2022). This highlights a need for resources and interventions to empower parents in approaching RES. Existing interventions—BPSS-RS, CounterACT, and EmBARK—demonstrate effective strategies for increasing parents' RES knowledge, confidence, and alignment between values and actions (*Black Parenting Strengths and Strategies-Racialized Short [BPSS-RS]* Coard et al., 2021; *Anti-Racist Consciousness Training [CounterACT]* Heberle et al., 2024; *Empowering Behaviors to Address Race with Kids [EmBARK]* Scott et al., 2024). Because these programs are not yet widely disseminated or applicable across racial-ethnic groups, key components (e.g., psychoeducation on children's racial development, motivational interviewing to address ambivalence) could be integrated into settings where parents already interface with professionals such as primary care, schools, and religious and community organizations. Such integration may help address parents' common concerns that children are “too young” for conversations about race and improve their ability to respond effectively during racialized events.

The final theme captured the ways in which children's environments shaped their parents' RES strategies, children's racialized experiences, and their language exposure. Parents across groups responded through proactive socialization strategies, environmental shaping, and reliance on broader networks to challenges imposed by environments outside of their control. Parents specifically emphasized the critical role of the school environment in shaping their children's RES. Latine parents often relied on schools not only for language socialization (Chaparro, 2019; Delgado-Gaitan, 1993; Melzi et al., 2023; Ochoa et al., 2023) but also for managing school-based conflicts related to race and racism (Anderson et al., 2015). Similarly, Black parents held expectations that African American history would be adequately covered in the curriculum and used family engagement at school as a preparation for bias strategy (Suizzo et al., 2008). Black families were also more likely to intervene at school to address issues of race and racism. Recent restrictions on discussions related to critical race theory may significantly limit children's exposure to diverse historical perspectives and reduce teachers' abilities to effectively address issues of race and racism. These changes will likely require more effort from parents across all racial-ethnic groups to engage in RES practices at home, and for minoritized parents to continue to intervene when their children experience racial conflict at school.

## 5 Strengths and limitations

A strength of this review is the conceptualization of parental RES as a contextually situated and dynamic process. Parents described not only oral communication related to RES but also highlighted more nuanced family interactions (e.g., discipline, routines, and modeling) used to convey RES messages. Parental endorsement of behavioral methods not currently captured in the literature lends support to the argument that RES is not independent from parenting but is simply part of the broader parenting process and that parents attempt to use developmentally appropriate methods during the early childhood period (Dunbar et al., 2017; Karam, 2024). Another strength is the inclusion of studies that sampled caregivers beyond mothers. These studies revealed meaningful differences in RES across caregivers—for instance, fathers sometimes used distinct strategies or were viewed as key racial socializers for boys—illustrating the value of moving beyond a mother-centric perspective to better understand family-level RES dynamics.

In addition to these strengths, several limitations should be noted. First, the use of only one coder during the article selection is a limitation of this study. Despite the use of predetermined inclusion/exclusion criteria and the first author's consultation with the second author during study screening and selection, study selection may have been influenced by subjective judgment, reducing the reproducibility of the screening process. Second, although some studies included multiple caregivers, the broader literature remains heavily reliant on maternal report, limiting generalizability of findings to fathers and other socialization agents and potentially underrepresenting important familial RES processes. Other limitations of the literature that undermine generalizability of this review are the overrepresentation of Black families and underrepresentation of Asian and Multiracial families, and the fact that the literature spans nearly two decades, during which major sociopolitical events (e.g., Black Lives Matter and the murder of George Floyd) may have shaped parental RES practices in ways that aggregated findings obscure.

## 6 Conclusion

Results of this meta-ethnographic review of the qualitative literature examining parental racial-ethnic socialization during early childhood highlight the complex and dynamic nature of this parenting process. Specifically, parents' own life experiences influence their values, beliefs, and intentions for RES with their children. These intentions lead to methods and messages of RES that are often closely related. The methods and messages parents utilize are frequently complicated by competing beliefs prompted by their children's traits, experiences, and environment. The resulting synthesis supports previous assertations that RES during early childhood is embedded within broader parenting practices and family processes, and highlights gaps and limitations of the literature that future research should seek to address such as a lack of diversity in samples (both in terms of family structure and race-ethnicity).

## Data Availability

The original contributions presented in the study are included in the article/supplementary material, further inquiries can be directed to the corresponding author.
